# SUV_fdg_: A standard-uptake-value (SUV) body habitus normalizer specific to fluorodeoxyglucose (FDG) in humans

**DOI:** 10.1371/journal.pone.0266704

**Published:** 2022-04-21

**Authors:** Bradley J. Beattie, Tim J. Akhurst, Finn Augensen, John L. Humm

**Affiliations:** 1 Department of Medical Physics, Memorial Sloan Kettering Cancer Center, New York, NY, United States of America; 2 Division of Radiation Oncology and Cancer Imaging, The Peter MacCallum Cancer Centre, Melbourne, Australia; Mayo Clinic, UNITED STATES

## Abstract

**Purpose:**

To devise a new body-habitus normalizer to be used in the calculation of an SUV that is specific to the PET tracer ^18^F-FDG.

**Methods:**

A cohort of 481-patients was selected for analysis of ^18^F-FDG uptake into tissues unaffected by their disease. Among these, 65-patients had only brain concentrations measured and the remaining 416 were randomly divided into an 86-patient test set and a 330-patient training set. Within the test set, normal liver, spleen and blood measures were made. In the training set, only normal liver concentrations were measured. Using data from the training set, a simple polynomial function of height and weight was selected and optimized in a fitting procedure to predict each patient’s mean liver %ID/ml. This function, when used as a normalizer, defines a new SUV metric (SUV_fdg_) which we compared to SUV metrics normalized by body weight (SUV_bw_), lean-body mass (SUV_lbm_) and body surface-area (SUV_bsa_) in a five-fold cross-validation. SUV_fdg_ was also evaluated in the independent brain-only and whole-body test sets.

**Results:**

For patients of all sizes including pediatric patients, the normal range of liver ^18^F-FDG uptake at 60 minutes post injection in units of SUV_fdg_ is 1.0 ± 0.16. Liver, blood, and spleen SUV_fdg_ in all comparisons had lower coefficients of variation compared to SUV_bw_ SUV_lbm_ and SUV_bsa_. Blood had a mean SUV_fdg_ of 0.8 ± 0.11 and showed no correlation with age, height, or weight. Brain SUV_fdg_ measures were significantly higher (P<0.01) in pediatric patients (4.7 ± 0.9) compared to adults (3.1 ± 0.6).

**Conclusion:**

A new SUV metric, SUV_fdg_, is proposed. It is hoped that SUV_fdg_ will prove to be better at classifying tumor lesions compared to SUV metrics in current use. Other tracers may benefit from similarly tracer-specific body habitus normalizers.

## Introduction

Standard clinical Positron Emission Tomography (PET) systems typically measure mean radioactivity concentration with a consistency on the order of about 2.5%, this limited primarily by the PET calibration process and the stability of the camera over time [1]. However, radioactivity concentration per se is often *not* a useful metric owing to its variation with the radioactivity of the injected dose. In order to monitor a tumor’s uptake of ^18^F-FDG over several weeks or months, for example, it is necessary to normalize the PET radioactivity concentration by the dose injected at each session, converting it into units of percent injected dose per milliliter (%ID/ml). Meaningful use of this metric assumes a degree of stability of the patient’s bodily systems between measurements, consistent timing of the measurement post injection, and linearity of the tissue uptake with injected dose within the range of doses administered (i.e. doubling the injected dose, doubles the tissue concentrations).

While %ID/ml is useful for intra-subject comparisons, it does not allow for meaningful comparisons between patients because it does not account for the variation in tissue uptake of ^18^F-FDG as a function of the patient body habitus. Larger patients tend to have lower %ID/ml concentrations because the radioactivity is distributed into a larger volume. Thus, to facilitate comparisons of tissue uptake across patients, an additional normalization is necessary. If the radiotracer distribution were to be essentially uniform within the body, then the appropriate additional normalizer would be the patient’s body mass (i.e. doubling the patient’s size, halves the tissue concentrations). And indeed, this is the normalizer, SUV_bw_ (see Eq 1), that is used most frequently.


$${SUV}_{bw}=\frac{tissue\;concentration(\frac{Bq}{ml})(\frac{ml}{g})}{injected\;dose(Bq)/patient\;weight(g)}$$ (1)

Although the SUV_bw_ metric is, to this day, widely employed, its deficits have frequently been raised, and at no time more strongly than by Keyes who in 1995 [2] concluded that it was a “silly useless value”. Most of Keyes’ objections could easily be addressed (e.g. by fixing the uptake period) or were not really about the SUV metric itself (e.g. partial volume effects) but at least for ^18^F-FDG (and likely for many other radiotracers) he correctly pointed out that interpatient differences in body composition and habitus are not well described by a linear function of body mass alone.

The need for a body habitus normalizer other than body weight stems from the fact that ^18^F-FDG does not distribute equally into all the normal tissues. On a per unit mass basis, uptake into adipose tissues (in particular), is much less than most other tissues. Thus, two subjects of identical mass but one having a larger fraction of that mass in the form of adipose reserves, will tend to have larger SUV_bw_ values in all their tissues.

Following this reasoning, Zasadny and Wahl in 1993 proposed that FDG uptake be normalized by lean-body mass (SUV_lbm_) and showed that SUV_bw_ measures of normal blood, liver and spleen all retained a strong correlation with body weight, whereas for SUV_lbm_, this correlation was greatly diminished. In a similar vein, Kim et al proposed in 1994 [3] normalizing instead by patient body surface-area (SUV_bsa_) and likewise showed reductions in liver correlation with weight. In neither of these studies was the patient body habitus normalizer (i.e. the actual lean-body mass or body surface-area) measured directly. Instead the normalizer was estimated using simple functions of height and weight, with the lean-body mass estimate making use of two separate functions, one for males and one for females. Kim et al [4] later went on to directly compare SUV_lbm_ to SUV_bsa_ concluding that SUV_bsa_ was superior based upon its relative lack of correlation to body habitus metrics. Nevertheless, in 2009, Wahl et al. [5] incorporated SUV_lbm_ (a.k.a. SUL) into their PERCIST criteria (the PET equivalent to the CT-based RECIST criteria) proposing it to be used as *the* standard for the evaluation of tumors using ^18^F-FDG.

Debate over these SUV metrics has continued through to the present day [6], much of this highlighting the vagaries of the lean-body mass and body surface-area estimates [7, 8] each of which can be calculated with one of several different formulas, while others have proposed various means of direct measurement of lean-body mass or body surface area [9–12] or other ancillary corrections [13, 14]. Despite these cogitations and the evidence suggesting that either SUV_bsa_ or SUV_lbm_ would be a better choice, SUV_bw_ remains as the most commonly reported metric in the literature and likely also in clinical use.

In the following, we propose to take a slightly different tact in addressing this question, recognizing that SUV_bw_, SUV_lbm_, SUV_bsa_ are all simply functions of patient height, weight and sex, and that maybe none of these surrogates is the optimal body habitus normalizer for ^18^F-FDG. Based on this premise, we will seek to devise a completely new normalizing function, one that is specific to ^18^F-FDG. As was the case in the previous evaluations of SUV metrics, we will assume that ^18^F-FDG uptake in a normal liver does not itself vary systematically with body habitus, age or sex. Moreover, we assert that confounding factors of any sort can only increase an SUV metric’s coefficient of variation (CoV) above the liver’s true normal range and thus smaller CoV values are indicative of a less biased normalizer.

## Materials and methods

### Patients

The data used in this study was derived from patients receiving standard of care ^18^F-FDG scans at our institution, mostly for the diagnosis and monitoring of cancerous lesions. Patients were excluded if they were diagnosed with a non-solid tumor type, had extensive disease, had any indication of lesions within an organ being measured or were imaged outside of the 55–75 minute post injection time window recommended by the European Association of Nuclear Medicine (EANM) [15] and the Quantitative Imaging Biomarkers Alliance (QIBA) [16]. A total of 481 patients meeting these criteria were included in the study. A subset of these (100 in all) were specifically sought after, selected based on their age (15 or under) in order to enrich the sample with smaller sized subjects.

Of the 481 patients, 65 had only their normal brain ^18^F-FDG uptake measured. The remaining 416 patients were randomly divided into a 330 subject training group that received only normal liver ^18^F-FDG uptake measurements and an 86-member test group within which normal liver, spleen and blood concentrations were measured. Of the 330 training group members, 153 were adult women, 116 were adult men and 61 were pediatric patients (note–here the division between pediatric and adult was taken to be 12 years of age, i.e. “adults” > 12 y). Within the test cohort there were 45 adult women, 31 adult men and 10 pediatric patients. And within the brain-only cohort, there were 14 adult women, 29 adult men and 22 pediatric patients.

Subjects were included regardless of what PET scanner model was used, so the cohort includes a mixture of scans from various GE PET cameras including Discovery PET/CT models DST, DSTE, D600, D690, D710, 3-ring DMI, 5-ring DMI and a Signa PET/MR. This data was analyzed under the auspices of a retrospective research protocol “Image Processing Applications for Medical Imaging Workstations and Systems”, IRB# 16-1488A(12), which was approved by the Memorial Sloan Kettering Cancer Center Institutional Review Board as Exempt, and the requirement for consent was waived.

### Measurements

Within the training and test cohort patient scans a single large region of interest (ROI) representing a volume of approximately 14 ml, was drawn well away from the diaphragm. Within the test cohort scans, additional ROIs were placed over homogenous regions well within the descending aorta (~1 mL) to measure the blood concentration, and spleen (~2.5 mL). Within scans of the brain-only test cohort, a single ROI was placed over a frontal grey matter region (~0.5 mL). In all cases the ROI was drawn free-hand on a single slice encompassing over a region that was homogeneous in its FDG uptake and (based on visualization of the entire tissue) representative of the tissue as a whole. All were drawn on transaxial images except for the descending aorta region which was drawn on a sagittal slice extending vertically along the aorta’s length and referencing the co-acquired CT. In addition to the mean radioactivity concentration within these regions, the following measures describing the patient scan were compiled: patient age at time of scan, weight, height, sex, injected radioactivity and the time interval between the injection and when the bed position over a measured region was acquired. All radioactivity concentration measures were appropriately decay corrected and divided by the injected activity to arrive at units of %ID/ml. This value was then multiplied by the patient’s body weight in grams, which if one assumes 1 g/ml, results in unitless SUV_bw_ values. The values were also multiplied by the calculated body surface-area and lean-body mass to arrive at SUV_bsa_ and SUV_lbm_ measures, respectively; making use of the body-surface area estimation function proposed by Du Bois [17] and the lean-body mass function used by Lodge and Wahl for PERCIST [18].

### Model development

In seeking an empirical functional form that would well describe the relationship between the liver mean %ID/ml and body habitus, we first reasoned that these two quantities should be roughly inversely proportional and therefore chose to attempt to model the multiplicative inverse of the liver %ID/ml (i.e. its mean concentration in units of ml/%ID). Moreover, since it was our preference that our model achieve specifically a high *percent* accuracy and result in only positive normalizing values, we chose to fit its log values (i.e. log[ml/%ID]).

Through some experimentation with the training set, least squares fits of various functions were compared [Curve Fitting Toolbox v 3.5.11, The MathWorks, Inc.] and a subjective “best” was selected making use of Bayesian (BIC) [19] and Akaike information criteria (AIC) [20], the adjusted R-squared value [21] of the fits and a visual examination of the residuals.

### Model validation and testing

Using the selected fitting function model, the training set was then entered into a 5-fold cross-validation study. In this study the training set was first randomly divided into five subgroups each containing 20% (i.e. 66) of the patients. Each of the 5 groups was then, in turn, used as a validation set, with the remaining 80% (264 patients) used to train (i.e. fit) the model. In each of the five validations, CoVs and correlations to height and weight for each of the four SUV metrics (SUV_bw_, SUV_lbm_, SUV_bsa_ and SUV_fdg_) were calculated and based on these numbers the performance of our proposed body habitus normalizing (BHN) function was assessed.

Following this validation, a single fitting procedure using the selected BHN model was applied to the entire 330 patient training set to determine its parameter values. This BHN function was then used to calculate the SUV_fdg_ values for all the normal tissue measurements taken from the two test sets. As was done in the cross-validation, SUV_bw_, SUV_lbm_ and SUV_bsa_ values were determined and compared based on their CoVs and correlations to height and weight, but in addition, for the test cohorts, correlation to age was also tested.

### Statistics

For every test of a linear relationship between a variable (SUV, residual, etc.) to patient height, weight or age, a Pearson’s correlation coefficient R and associated *P* value were determined. This *P* value indicates the probability of seeing a sample correlation coefficient of that magnitude when the true population correlation is zero and was calculated using two tails of a t-distribution with n-2 degrees of freedom (where n is the number of samples) after first converting the R value to a t-statistic using the formula $t=\frac{R\sqrt{n-2}}{1-r^{2}}$. In all cases significance was assessed at an alpha level of 0.05, corrected for multiple comparisons following Bonferroni [22] where indicated. The comparison of adult and pediatric brain SUV_fdg_ values was made with an unpaired two-tailed, two sample *t*-test assuming unequal variances.

## Results

### Cohort characterization

Subjects ranged from 9 months to 91 years of age and were roughly evenly distributed over this range (see S1 Fig) owing to the enriched selection of pediatric patients. Women tended to be smaller in both their height, 1.62 ± 0.07 m, and weight 70 ± 19 kg, compared to the men who tallied in at 1.74 ± 0.09 m and 83 ± 20 kg, respectively. Pediatric patients (under 12) averaged 1.17±0.20 m in height and weighed 23 ± 11 kg.

### Model development

**Fig 1:**
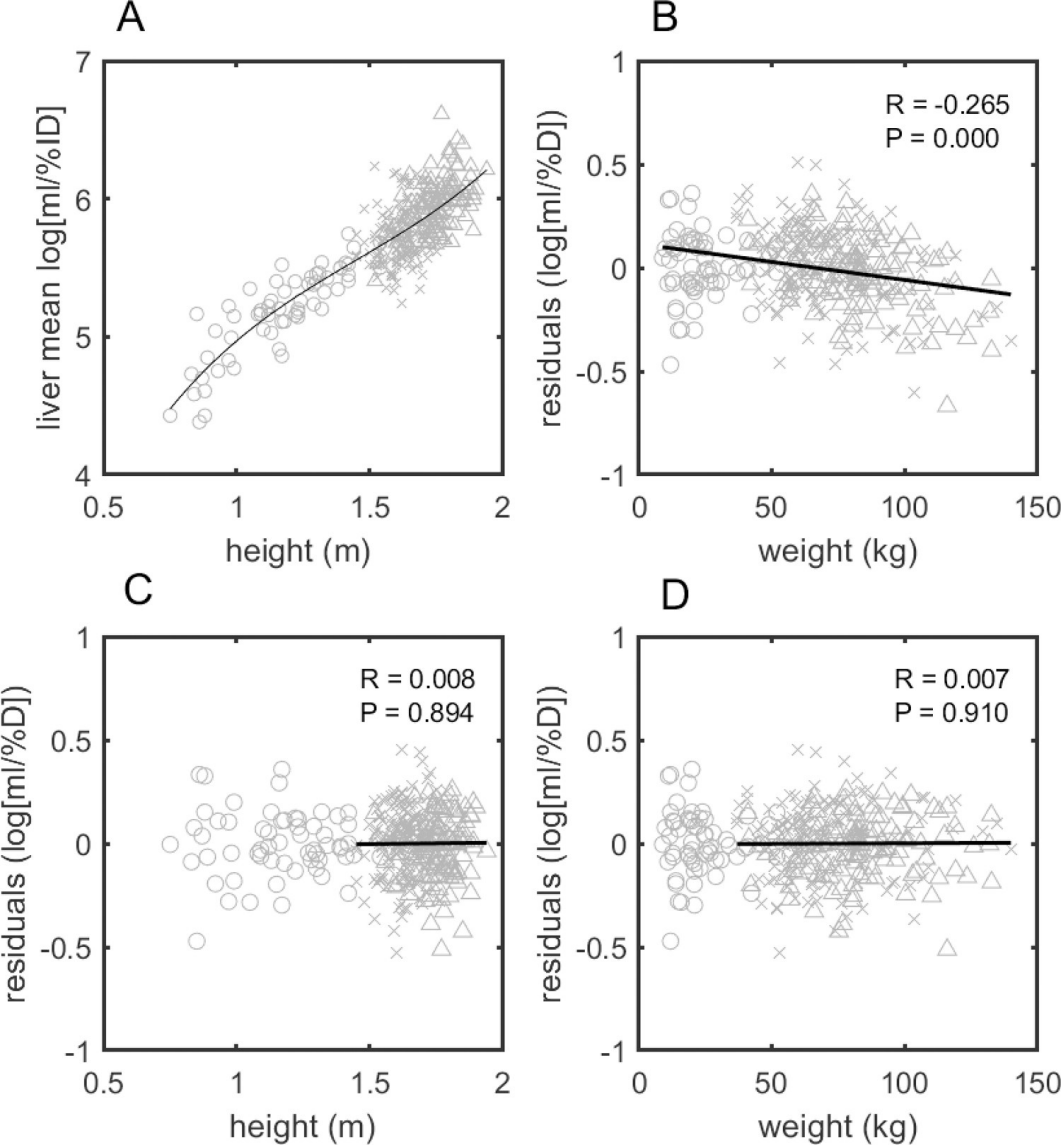
Training data, liver mean fits and residuals as a function of height and weight. Scatter plots showing the fit of the training set data to a third-order polynomial function of height (A), the residuals of that fit as a function of weight (B), the residuals of the model A fit to the log of the mean liver ml/%ID as a function of height (C) and its residuals as a function of weight (D). In all graphs, triangles depict male patients, x’s refer to female patients and o’s are children under the age of 12. The fits of the residuals shown in (C) and (D) along with the associated correlation coefficients and *P* values shown in the legend, excluded patients under the age of 12 so that these patients wouldn’t have outsized influence over the correlation. For Model A, regardless of whether the pediatric patients were included or not, there was no significant correlation of the residuals to either height or weight.

Using data from the training set only, visual inspection of the weight vs. log(ml/%ID) and height vs. log(ml/%ID) suggested that simple functions of weight did not fit the data well (see S2 Fig) whereas a third order polynomial function of *height* estimated the log(ml/%ID) values in an unbiased manner (see Fig 1A). As a means of confirming this, second and fourth order polynomial functions of height were also tried and compared based on AIC, BIC and adjusted R^2^ values (see Table 1). The AIC and adjusted R^2^ values showed a slight preference for the 3^rd^ order model, but the BIC was best for the 2^nd^ order polynomial function, therefore both models remained under consideration.

**Table 1:** Model information criteria evaluation.

	2nd order	3rd order	4th order	Model A	Model B
AIC	-1110	-1111	-1110	-1185	-1176
BIC	-1099	-1096	-1091	-1166	-1161
adjR^2^	0.725	0.727	0.726	0.782	0.775

Akaike and Bayes Information Criteria and Adjusted R^2^ values for fits of various models to log(ml/%ID) of the training cohort. The 2^nd^, 3^rd^ and 4^th^ order models are a function of height alone. Models A and B are functions of height and weight.

Although functions of weight alone did not appear to predict the liver concentrations well, there was still a potential that the addition of height information might improve the fit substantially. Therefore, we added a linear term incorporating height to the 3^rd^ order function of weight (see Model C in Eq 2). However, the fit continued to be poor, especially for small patients (see S3 Fig) and so we dropped Model C from further consideration.

Then to ascertain whether adding weight information might improve the estimate of the 3^rd^ order function of height model, we plotted its residuals as a function of patient weight (see Fig 1B). This plot showed that there was remaining correlation which could perhaps be improved if weight were to be incorporated. This potential also remained for the 2^nd^ order function of height, so to each of these models was added a single parameter, *d*, incorporating the weight information. We will hereafter refer to the 3^rd^ order height plus 1^st^ order weight function as Model A and the 2^nd^ order height plus 1^st^ order weight as Model B (see Eq 2). Adding weight information in this way to Model A removed all correlation of the residuals with either height or weight (see Fig 1C and 1D, respectively).

$$\begin{array}{l} log[ml/\%ID]=ax^{3}+bx^{2}+cx+dy+e\\ \mathrm{where}\;\mathrm{for}\;\mathrm{model}\;A:\;x=height\;and\;y=weight\\ \mathrm{for}\;\mathrm{model}\;C:\;x=weight\;and\;y=height\\ \mathrm{for}\;\mathrm{model}\;B:\;a=0,\;x=height\;and\;y=weight \end{array}$$ (2)

Model A and Model B were both fit to the training set data. In this instance, however, the AIC, BIC and adjusted R^2^ values (again see Table 1) were all better for model A. The residuals for Model A’s fit to the data are plotted as functions of height and weight in Fig 1C and 1D, respectively. In these plots we emphasize that there remains no correlation to body habitus even when restricted to the adult population by showing the fit based only on those patients. The correlation remained near zero, however, when the pediatric patients were included. Based on these assessments we selected Model A as the functional form to be used as the BHN when calculating SUV_fdg_.

### Model testing

Using model A as the functional form for our proposed BHN, a 5-fold cross validation assessment comparing SUV_fdg_ to SUB_bw_, SUV_lbm_ and SUV_bsa_ was conducted using data in the training set. In this assessment, the data was randomly partitioned into 5 validation groups each containing 66 patients. Then in turn for each of these groups, the remaining 264 patients were used to fit the five parameters of model A. The means and standard deviations for each of the coefficients over the five fits were as follows: a = 1.46 +/- 0.13, b = -6.48 +/- 0.56, c = 10.10 +/- 0.74, d = 0.00512 +/- 0.000226, e = -0.168 +/- 0.312. The resulting normalizing function was then applied to calculate SUV_fdg_ values (see Eq 3) for the validation group along with values for SUV_bw_, SUV_lbm_, and SUV_bsa_.


$${SUV}_{fdg}=\frac{tissue\;concentration(\frac{Bq}{ml})}{injected\;dose\;(Bq)/BHN(ml)}=\frac{\%ID}{ml}∙\frac{BHN(ml)}{100}$$ (3)

Coefficients and *P* values were calculated for each SUV’s correlation to height and weight. The *P* values were each assessed at an alpha level of 0.05 but Bonferroni corrected [22] for the 5 tests (i.e. were considered significant at *P* values < 0.01). CoVs for each of the SUVs were also determined. The results of this assessment are shown in Table 2. The mean of the five SUV_fdg_ CoVs, 0.16, was taken to be the best estimate of the population normal liver SUV_fdg_ standard deviation and was reported in the Abstract. In all five validations, SUV_fdg_ had the smallest CoV, followed by SUV_bsa_, then SUV_lbm_ with SUV_bw_ having the largest CoV. This same pattern was seen in the correlation coefficients describing the relationship of the SUV metrics to both patient height and weight, with SUV_fdg_ always showing the lowest correlation. In five out of five tests, the correlation coefficients to height and weight for SUV_bw_ and SUV_lbm_ were significant. In one out of five tests the correlation of SUV_bsa_ to height was significant, but no correlations of SUV_bsa_ to weight occurred. In no instance was the correlation of SUV_fdg_ to either height or weight found to be significant.

**Table 2:** Cross validation results.

	statistic	SUVbw	SUVlbm	SUVbsa	SUVfdg
	CoVs	0.289 ± 0.010	0.223 ± 0.015	0.175 ± 0.014	0.162 ± 0.014
weight	R-value	0.768 ± 0.051	0.523 ± 0.020	0.217 ± 0.019	-0.000+/-0.068
	*P* value	0.000 ± 0.000	0.000 ± 0.000	0.083 ± 0.030	0.695 ± 0.216
	N of 5 sig	5	5	0	0
height	R-value	0.602 ± 0.096	0.623 ± 0.062	0.234 ± 0.064	-0.008 ± 0.096
	*P* value	0.000 ± 0.000	0.000 ± 0.000	0.082 ± 0.065	0.541 ± 0.194
	N of 5 sig	5	5	1	0

Results from the 5-fold cross validation comparing SUV_bw_, SUV_lbm_, SUV_bsa_ and SUV_fdg_ based upon the coefficients of variation (smaller is better) and correlations to height and weight (again, smaller is better). The “N of 5” rows indicate the number of times a correlation coefficient was determined to be significant.

### BHN parameter determination

Model A’s final parameter values were determined in a single least-squares fit of the entire 330 patient training set and are described in Eq 4, wherein height is measured in meters, weight in kilograms and the coefficients are all taken to have units such that the unit of the final BHN result is in milliliters. Coefficient values are shown, followed by the 95% confidence interval in parenthesis.

$$\begin{array}{l} \;BHN=100e^{\left(ax^{3}+bx^{2}+cx+dy+e\right)}\\ \mathrm{where}:\;x=height\;and\;y=weight\\ \;a=2.03\;(1.13,2.93)\\ \;b=-9.07\;(-12.92,-5.22)\\ \;c=13.94\;(8.56,19.33)\\ \;d=0.00539\;(0.00438,0.00641)\\ \mathrm{and}:\;e=-2.04(-4.49,0.41) \end{array}$$ (4)

**Fig 2:**
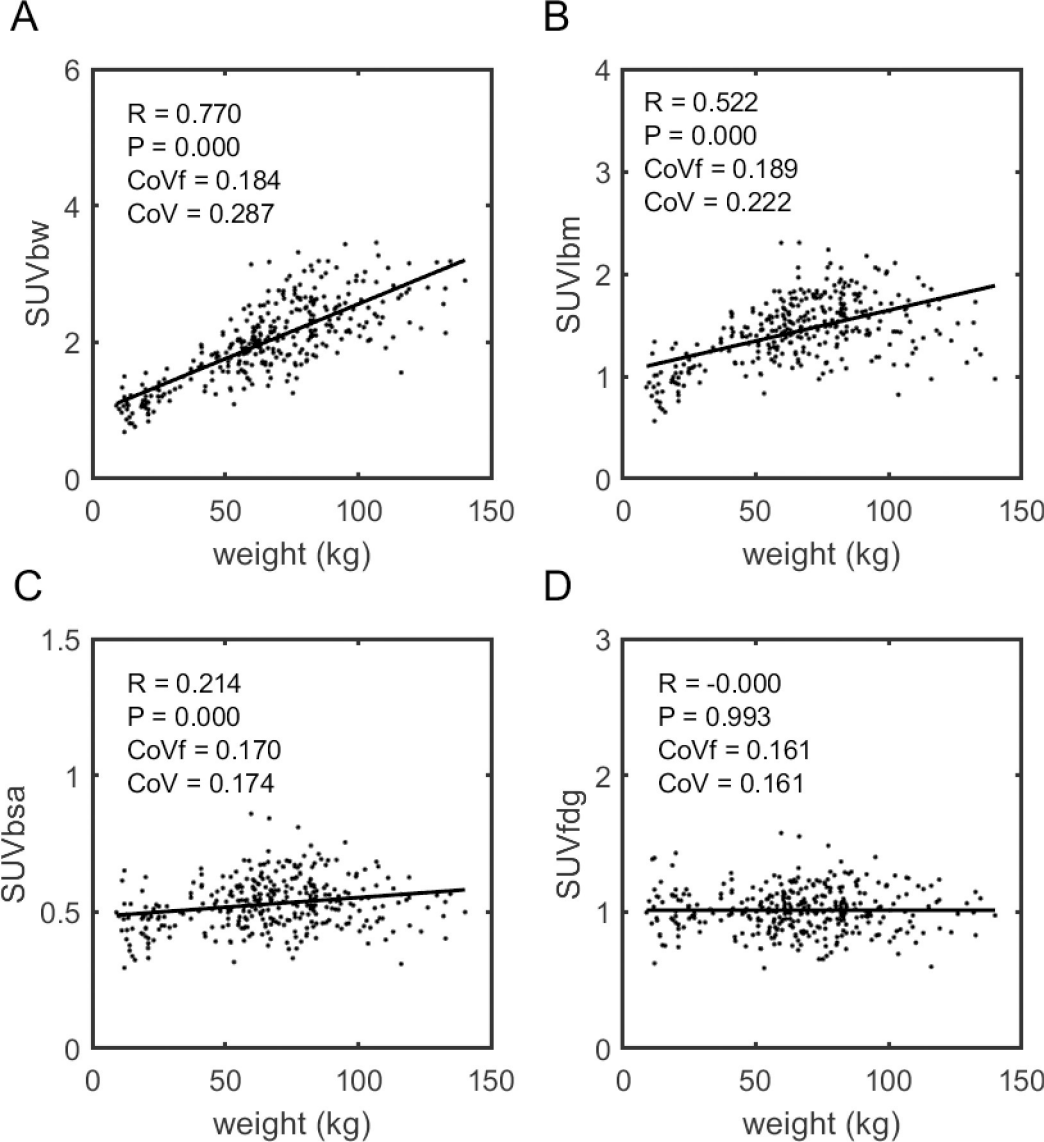
Training data, SUVs as a function of weight. Scatter plots showing the correlation of various types of liver SUV measurement to patient weight within the training cohort, (A) SUV_bw_, (B) SUV_lbm_, (C) SUV_bsa_ and (D) the proposed SUV_fdg_ metric. Only SUV_fdg_ shows no significant correlation to weight. The CoVf value describes the variance about the fitted line while CoV describes the variance about the mean.

**Fig 3:**
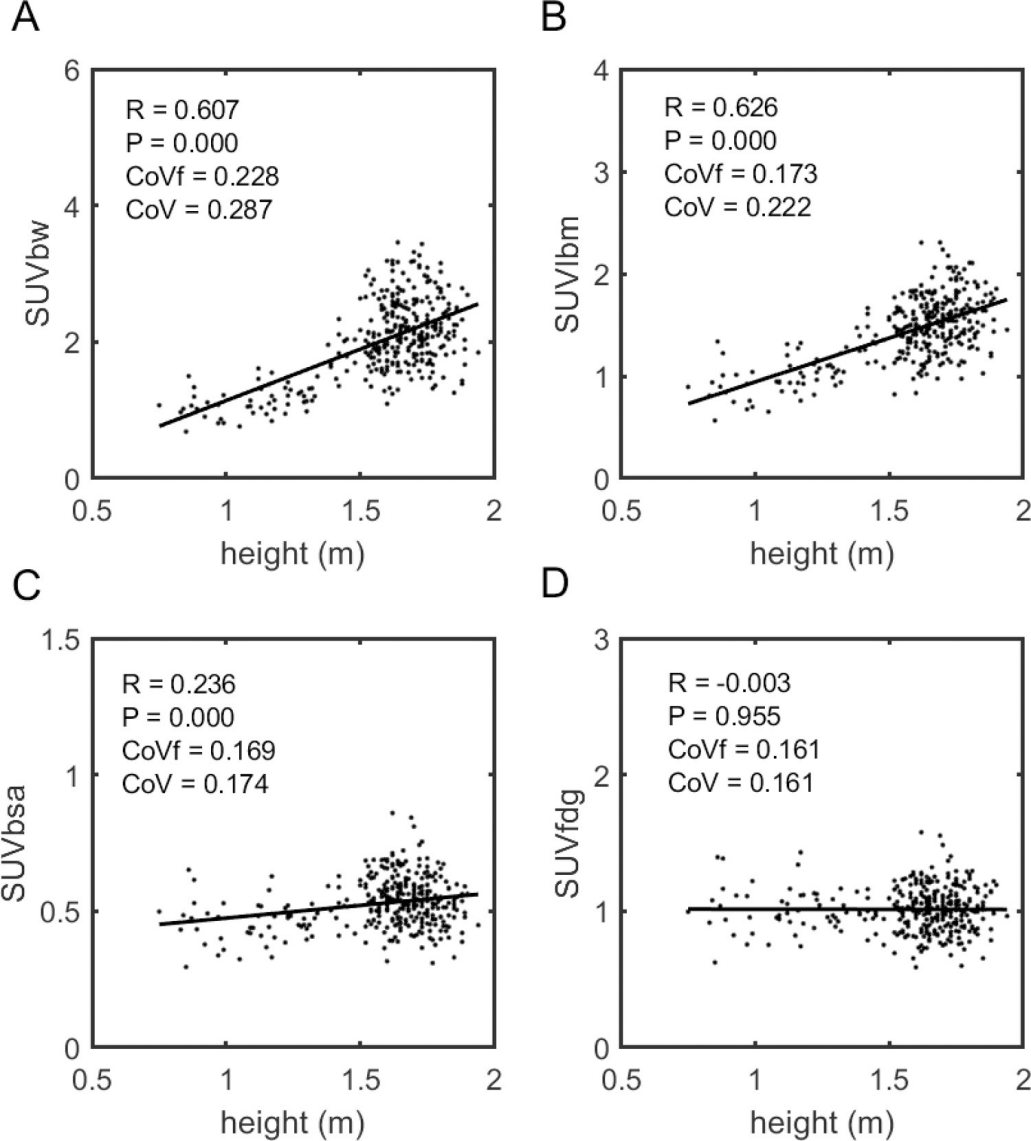
Training data, SUVs as a function of height. Scatter plots of liver measurements like those shown in Fig 2 except here plotted as a function of patient height. Again, only SUV_fdg_ has no significant correlation.

The quality of these fits to this function can be appreciated by viewing the 3D scatter plot showing the proposed BHN function surface on log[ml/%ID] vs. height vs. weight axes (S4 Fig). Moreover, Figs 2A–2D and 3A–3D show the correlations for the full training cohort to weight and height, respectively, of the standard SUV metrics (SUV_bw_, SUV_lbm_ and SUV_bsa_) in comparison to that of the SUV_fdg_ metric. The results here essentially recapitulate those of the 5-fold cross validation with SUV_bw_ and SUV_lbm_ both having clearly significant correlation to body habitus, SUV_bsa_ showing weak correlation, and SUV_fdg_ having no significant correlation.

### Test cohort results

**Fig 4:**
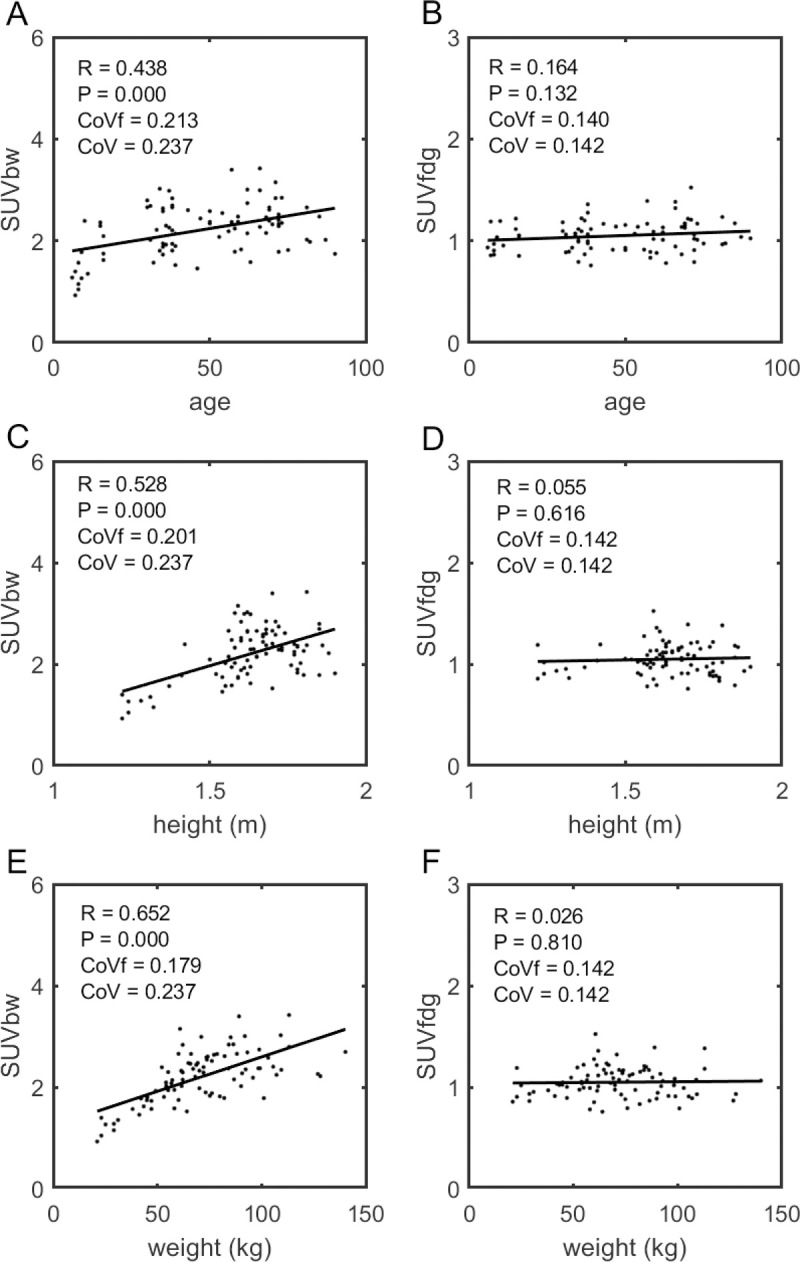
Liver test data, SUVs as a function of age, height and weight. For the independent test data, these scatter plots compare the correlations in liver SUV_bw_ (column A, C, E) and SUV_fdg_ (column B, D, F) measurements with age (row A, B), height (row C, D) and weight (row E, F).

**Fig 5:**
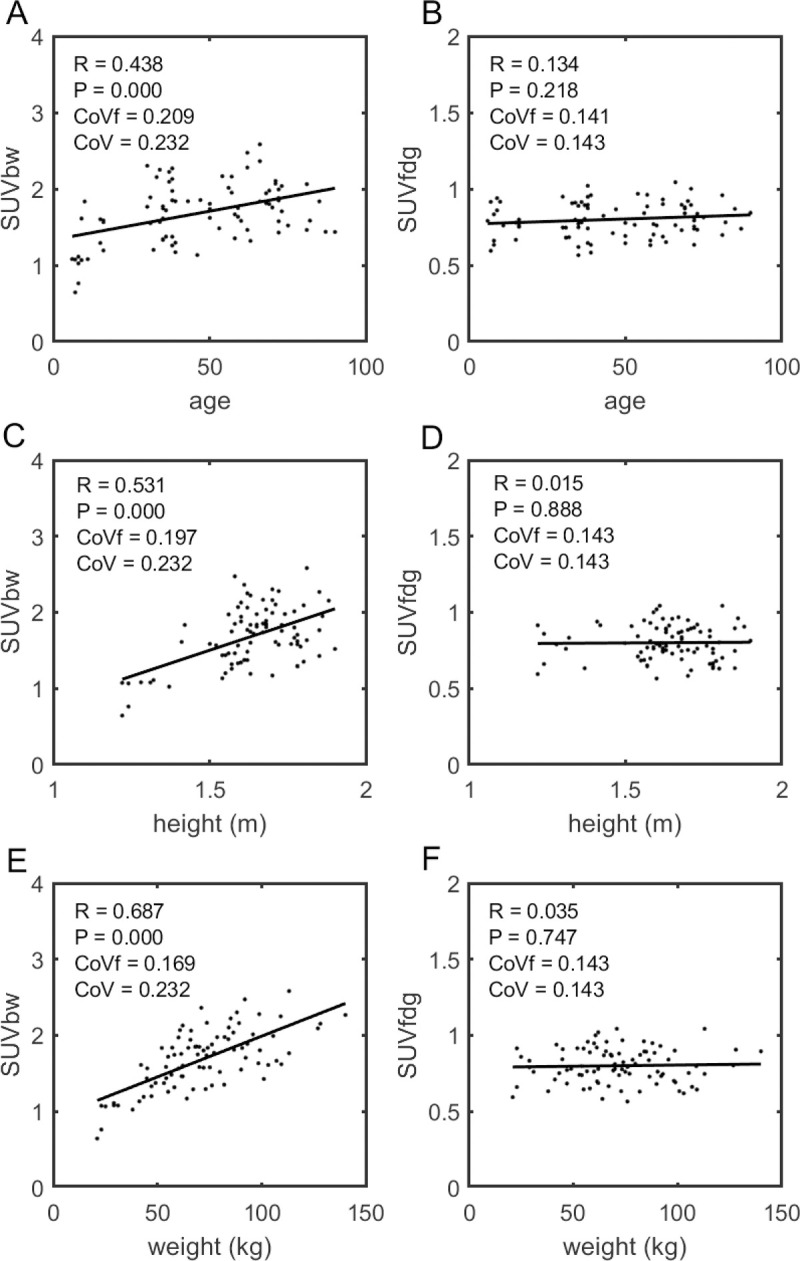
Blood test data, SUVs as a function of age, height and weight. For the independent test data, these scatter plots compare the correlations in blood SUV_bw_ (column A, C, E) and SUV_fdg_ (column B, D, F) measurements with age (row A, B), height (row C, D) and weight (row E, F). Note, blood concentrations were not measured in the training cohort and played no part in determining the BHN function used to calculate these SUV_fdg_ values.

The training set results for the liver were confirmed in the independent test cohort. Scatter plots show no significant correlation of SUV_fdg_ to either height or weight (see Fig 4). but also, no correlation to patient age, a parameter not considered in the determination of the BHN model. Importantly, these improvements also extended to tissues not at all used in the derivation of the BHN function. Scatter plots showing the correlations with height, weight and age for the normal spleen are shown in S5 Fig and for blood in Fig 5. That SUV_fdg_ appears to be a good predictor of the patient’s blood concentration (at this time post injection) is particularly significant given the relationship between the area under the blood time vs. activity curve (TAC) and absolute quantitative uptake of ^18^F-FDG.

Interestingly, measurements of gray matter uptake taken from the independent brain-only test cohort, show a small reduction in all four SUV metrics as a function of age in adult patients (see S6 Fig). These decreases did not reach statistical significance but are consistent with at least one other study which showed reduced brain glucose metabolic rates in older adults based on a modeled quantitative assessment of ^18^F-FDG uptake [23]. SUV_fdg_, SUV_bsa_ and to a lesser extent SUV_lbm_ all showed noticeably higher levels in the pediatric patients (< = 18 y) within this cohort. The SUV_fdg_ values for these two groups, 4.7 ± 0.9 for pediatric patients compared to 3.1 ± 0.6 for adults, were found to be significantly different (P<0.01) in a two-sample t-test. This was not the case, however, for SUV_bw_. All CoV and correlation results for all tissues measured in the independent test cohorts were also tabulated (see Table 3).

**Table 3:** Independent test cohort results.

			weight		height		age	
	statistic	CoV	R	P	R	P	R	P
LIVER	SUVbw	0.24	0.65	0.00	0.53	0.00	0.44	0.00
	SUVlbm	0.18	0.43	0.00	0.54	0.00	0.49	0.00
	SUVbsa	0.17	0.07	0.54	0.13	0.22	0.29	0.01
	SUVfdg	0.14	0.03	0.81	0.05	0.62	0.16	0.13
BLOOD	SUVbw	0.23	0.44	0.00	0.53	0.00	0.69	0.00
	SUVlbm	0.16	0.28	0.01	0.10	0.34	0.07	0.50
	SUVbsa	0.18	0.50	0.00	0.54	0.00	0.47	0.00
	SUVfdg	0.14	0.13	0.22	0.02	0.89	0.04	0.75
SPLEEN	SUVbw	0.23	0.61	0.00	0.41	0.00	0.34	0.00
	SUVlbm	0.16	0.35	0.00	0.38	0.00	0.39	0.00
	SUVbsa	0.16	-0.08	0.44	-0.13	0.23	0.09	0.39
	SUVfdg	0.16	-0.16	0.13	-0.26	0.01	-0.07	0.51
BRAIN	SUVbw	0.21	-0.15	0.41	0.16	0.37	-0.30	0.09
	SUVlbm	0.19	0.13	0.48	0.33	0.06	-0.11	0.53
	SUVbsa	0.19	-0.07	0.69	-0.06	0.73	-0.01	0.97
	SUVfdg	0.19	0.11	0.55	0.16	0.38	-0.11	0.55

Variance and correlation results for SUV_bw_, SUV_lbm_, SUV_bsa_ and SUV_fdg_ metrics for data measured from patients within the independent test cohorts. ^18^F-FDG concentrations were measured in normal liver, blood, spleen and brain. CoV is the coefficient of variation, R is the correlation coefficient and *P* is the probability of seeing an R value of that magnitude when the true population correlation is zero.

## Discussion

Herein we propose a new body habitus normalizer to be used when calculating SUV values within PET ^18^F-FDG patient studies. This body habitus metric, like the estimates of lean-body mass and body-surface area, is based on simple measures (height and weight) of the patient that can be determined prior to imaging. Like SUV normalized by body weight, the SUV_fdg_ metric calculated using the proposed normalizer can be considered unitless. The value can be interpreted as a fraction of the expected normal liver mean uptake at (approximately) 60 minutes post injection wherein normal liver is expected to have a value of 1.0 and therefore any tissue with an SUV_fdg_ value of 2.0, for example, has twice the liver’s uptake. As such, this metric can also be used as a quick quality assurance measure to identify data entry errors for the injected dose, its timing, or errors in the entry of the patient height or weight. Values dramatically different from 1.0 ± 0.16 measured in a normal region of a patient’s liver would be indicative of a problem, including perhaps significant extravasation of the injectate.

To the extent that the proposed BHN function can accurately predict the uptake to the normal liver and proportionately that of other normal organs (in units of %ID/g), this function may be useful in models seeking to estimate patient-specific radiation dose, thus allowing an a priori individualized assessment of the risk posed by the ^18^F-FDG injection. Similarly, this same information can be used in models of patient attenuation and scatter, which can then be combined with models of specific PET cameras to arrive at estimates of the expected noise equivalent count (NEC) rate for different body-parts. This information can then, in turn, be used to adjust imaging time to achieve a target image quality. Assuming the intrinsic resolution of most clinical PET cameras is about the same (or can be made so with appropriate smoothing) matching total effective NECs (factoring in the use of time-of-flight and the camera’s timing resolution) should go a long way towards harmonizing image quality across patients of different sizes and across institutions having a mixture of PET camera models.

Although SUV_fdg_ is specific to ^18^F-FDG PET, the concept behind it should be applicable to all tracers for which a suitable normal reference tissue can be found, and where any metabolism is either consistent or at least predictable across patients.

### Limitations

While in principle each radiotracer could/should have its own optimal body-habitus normalizer, this normalizer may not be well represented as a function of simple patient descriptors (height, weight, sex). For example, differences in tracer metabolism or excretion among patients could easily the dominant factor in determining tracer uptake without correlation to body-habitus. And even when some body-habitus metric could work, difficulty finding an appropriate reference normal tissue that is sufficiently large and low noise might hamper the ability to define a good normalizer.

While we have yet to directly demonstrate improved performance with SUV_fdg_ compared to other SUV metrics when applied to clinically relevant questions, we wish to point out that “improved performance” may be difficult to define in that it could mean either finding correlation where none was seen previously, or removing correlation with a clinical metric that was in truth dependent upon a parameter confounding the original SUV metric. In other words, if for example mean SUV_bw_ of a tumor was found to predict survival, it’s conceivable that this prediction was actually driven by patient body weight, largely or completely independent of FDG uptake in the tumor. In such cases, the improved performance of SUV_fdg_ might mean the spurious correlation would no longer be significant. Nevertheless, we will be applying SUV_fdg_ if future studies seeking to use FDG uptake as biomarker to seek correlations or divide tumors into subgroups. We expect that SUV_fdg_ will prove to be particularly useful in patient cohorts involving a wide range of patient sizes including potentially pediatric subjects.

The SUV_fdg_ metric we propose is no doubt imperfect. It is a function of just two parameters (patient height and weight) and was not found using an exhaustive search of potential functional forms. In no way do we claim that it is optimal. This we believe is in keeping with previously published SUV metrics. Normalization to reference tissues, or adjustments based on blood glucose measurements, or normalizations based on direct measurements of fat, muscle, and other normal tissue volumes, may all prove to be better than the metric we propose, in some contexts, however, in keeping with the spirit of SUV-type measurement, the metric we propose is applicable in all contexts, regardless of what body-parts are scanned and regardless of the availability of other refining variables. Moreover, we feel we have demonstrated (hopefully) convincingly that SUV_fdg_ is less confounded and has less variability than SUV_bw_, SUV_lbm_ and SUV_bsa_.

A key assumption when calculating and using this new body-habitus normalizer is that the rate constants governing normal liver ^18^F-FDG uptake are essentially the same (i.e. within a normal range) across all subjects regardless of age or sex. In other words, we have assumed that normal liver is itself a good normalizer, one whose uptake is proportional to the area under the curve of the arterial blood input function up until the time of the PET measurement at 60 minutes post injection. This assumption is strongly supported by our SUV_fdg_ measurements of the blood. As such, the proposed function should also be a useful normalizer for most other normal and abnormal tissues within the body.

One potential caveat to this assumption and possible confound in this study, is that we did not screen for fatty liver disease or other liver morbidities that might correlate with body habitus [24]. As such, the proposed BHN in its current form effectively includes estimations of the prevalence and impact of these disease processes in our population. Similarly, if liver ^18^F-FDG uptake in absolute terms varied significantly with age in the pediatric population, the proposed SUV_fdg_ metric would normalize away that difference given the high correlation between age and height in children. In other words, an SUV_fdg_ value of 1.0 can be considered to be the normal liver uptake level for pediatric subjects regardless of age even if the uptake in units of mg/min/100g were to go up or down as a function of age. Given our results in the blood, however, we feel these effects are at most, small.

When evaluating the performance of SUV_fdg_ relative to the other SUV types we have relied on two related assessments, each SUV metric’s CoV and the absence of any correlation to body habitus (specifically height and weight). The assessment based on correlation to body habitus has been used by others [3, 4, 25] but to our knowledge we are the first to make use of CoV for this purpose. In using CoV to assess SUV’s, we reasoned that the optimal SUV metric should accurately reflect the normal variation in liver ^18^F-FDG uptake and that any additional noise or confounds would only lead to increased CoV. This should be true so long as the variance in normal values, which presumably are randomly distributed about the mean, is not itself correlated with the SUV metric. It is anticipated that because of its reduced CoV and correlation to body habitus, it will likely outperform SUV_bw_, SUV_lbm_ and SUV_bsa_ when used to distinguish between two or more conditions, for example when classifying benign and malignant tumors across multiple patients.

## Conclusion

A new body habitus normalizer and associated SUV metric are proposed. This metric, SUV_fdg_, is intended to be used solely for the evaluation of the uptake of FDG and may in future studies be shown to outperform SUV metrics normalized by body weight, lean-body mass and body surface area.

## Supporting information

S1 Fig Age distribution of males and females in whole cohort. Histograms showing the distribution of ages for the entire cohort of 481 patients. The frequencies for males (A) and for females (B). These plots show that the sampling was roughly uniform with respect to patient age, a result achieved owing to an enhanced search for younger patients.
(PDF)

S2 Fig Poor fit of liver training data, log inverse concentration as function of weight alone. Scatter plot of training set data showing relationship between patient weight and mean liver concentration expressed in units of log(ml/%ID) and fitted with a 3^rd^ order polynomial. This fit performs poorly for subjects below 20 kg.
(PDF)

S3 Fig Poor fit of liver training data, log inverse concentration as function of both height and weight. Same data as shown in Figure S2 except now as a function of both height and weight and wherein the fit consists of a 3^rd^ order function of weight combined with a linear function of height. The addition of height in this case did *not* improve the fit for small subjects.
(PDF)

S4 Fig Good fit of liver training data, log inverse concentration as function of both height and weight. Three-dimensional scatter plot of data from the training set showing normal liver reciprocal mean concentration in units of ml/%ID shown on a log scale and plotted as a function of patient height and weight along with a surface showing the model A prediction.
(PDF)

S5 Fig Spleen test data, SUVs as a function of weight, height and age. For the independent test data, these scatter plots compare the correlations in normal spleen SUV_bw_ (column A, E, I), SUV_lbm_ (column B, F, J), SUV_bsa_ (column C, G, K) and SUV_fdg_ (column D, H, L) measurements with weight (row A, B, C, D), height (row E, F, G, H) and age (row I, J, K, L). Note, spleen concentrations were not measured in the training cohort and played no part in determining the BHN function used to calculate these SUV_fdg_ values.
(PDF)

S6 Fig Brain test data, SUVs as a function of weight, height and age. For the brain-only independent test data, these scatter plots compare the correlations in normal frontal gray matter SUV_bw_ (column A, E, I), SUV_lbm_ (column B, F, J), SUV_bsa_ (column C, G, K) and SUV_fdg_ (column D, H, L) measurements with weight (row A, B, C, D), height (row E, F, G, H) and age (row I, J, K, L). Note, brain concentrations were not measured in the training cohort and played no part in determining the BHN function used to calculate these SUV_fdg_ values. The lines and associated parameters seen in the legends were fitted to data from only the adult (>18 y) patients. The SUV_fdg_ values suggest a significant difference in brain glucose metabolism between adult and pediatric populations. In all graphs, triangles depict male patients, x’s refer to female patients and o’s are children under the age of 18.
(PDF)
