# Supplementary material for: SUVfdg: A standard-uptake-value (SUV) body habitus normalizer specific to fluorodeoxyglucose (FDG) in humans
Source: PLoS One. 2022 Apr 21;17(4):e0266704. doi: 10.1371/journal.pone.0266704 (PMC9022879; doi:10.1371/journal.pone.0266704)
Supplement: S1 Fig — Histograms showing the distribution of ages for the entire cohort of 481 patients. The frequencies for males (A) and for females (B). These plots show that the sampling was roughly uniform with respect to patient age, a result achieved owing to an enhanced search for younger patients. (PDF) [file pone.0266704.s001.pdf]

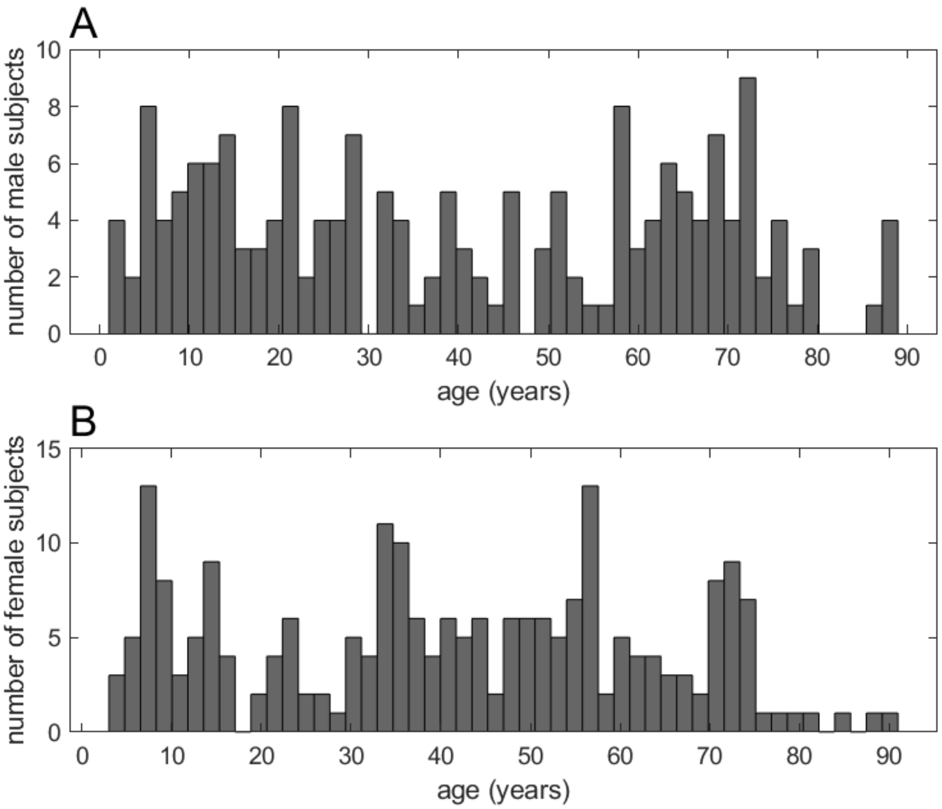

FIGURE S1. Histograms showing the distribution of ages for the entire cohort of 481 patients. The frequencies for males (A) and for females (B). These plots show that the sampling was roughly uniform with respect to patient age, a result achieved owing to an enhanced search for younger patients.
