# Supplementary material for: SUVfdg: A standard-uptake-value (SUV) body habitus normalizer specific to fluorodeoxyglucose (FDG) in humans
Source: PLoS One. 2022 Apr 21;17(4):e0266704. doi: 10.1371/journal.pone.0266704 (PMC9022879; doi:10.1371/journal.pone.0266704)
Supplement: S2 Fig — Scatter plot of training set data showing relationship between patient weight and mean liver concentration expressed in units of log(ml/%ID) and fitted with a 3rd order polynomial. This fit performs poorly for subjects below 20 kg. (PDF) [file pone.0266704.s002.pdf]

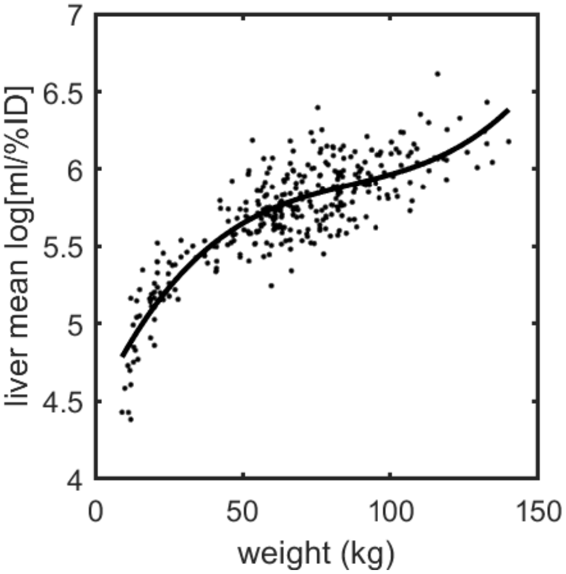

FIGURE S2. Scatter plot of training set data showing relationship between patient weight and mean liver concentration expressed in units of log(ml/%ID) and fitted with a 3rd order polynomial. This fit performs poorly for subjects below 20 kg.
