# Supplementary figures and images for: SUVfdg: A standard-uptake-value (SUV) body habitus normalizer specific to fluorodeoxyglucose (FDG) in humans
Source: PLoS One. 2022 Apr 21;17(4):e0266704. doi: 10.1371/journal.pone.0266704 (PMC9022879; doi:10.1371/journal.pone.0266704)

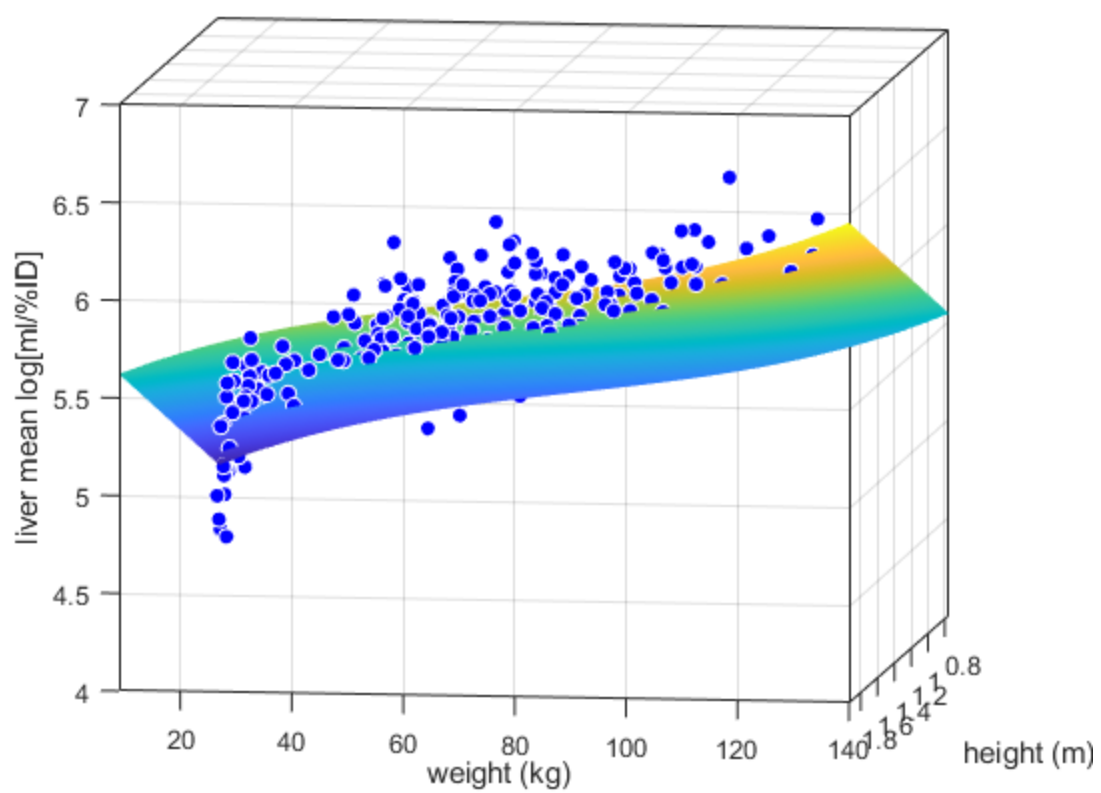

Supplement: S3 Fig — Same data as shown in Figure S2 except now as a function of both height and weight and wherein the fit consists of a 3rd order function of weight combined with a linear function of height. The addition of height in this case did not improve the fit for small subjects. (PDF) [file pone.0266704.s003.pdf]

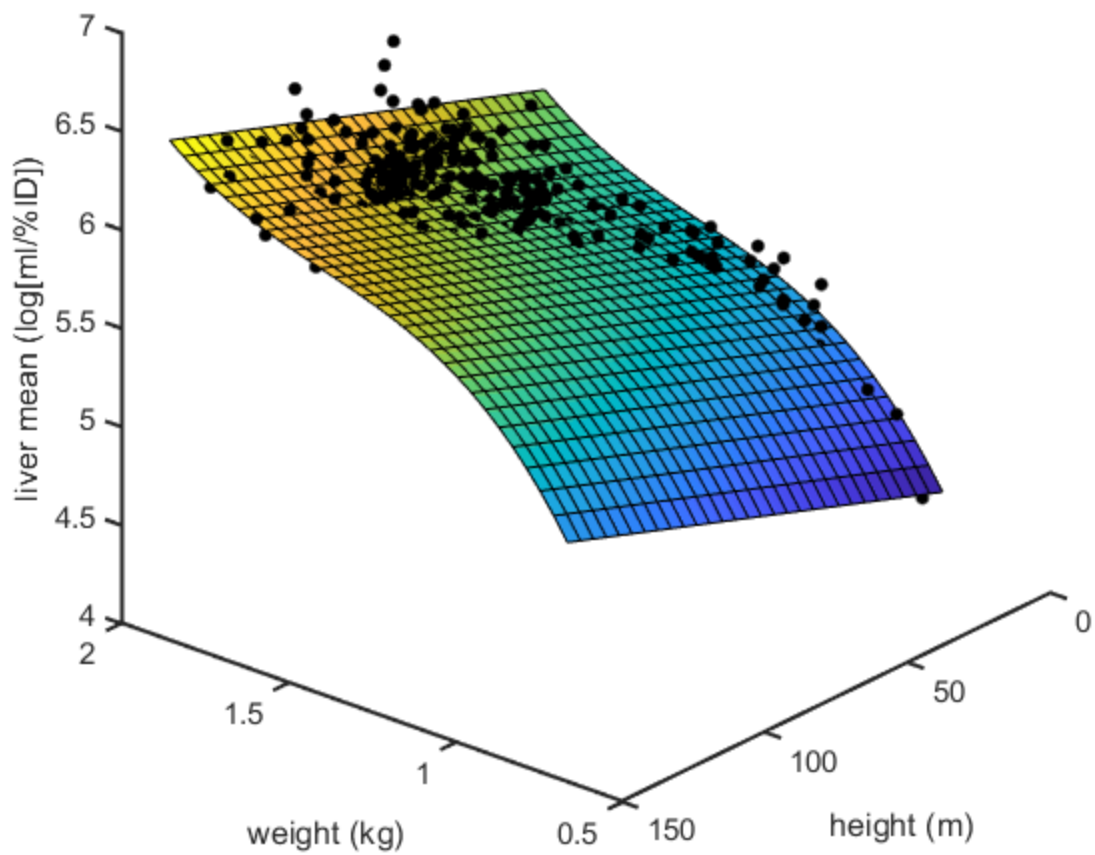

Supplement: S4 Fig — Three-dimensional scatter plot of data from the training set showing normal liver reciprocal mean concentration in units of ml/%ID shown on a log scale and plotted as a function of patient height and weight along with a surface showing the model A prediction. (PDF) [file pone.0266704.s004.pdf]
