# Supplementary material for: SUVfdg: A standard-uptake-value (SUV) body habitus normalizer specific to fluorodeoxyglucose (FDG) in humans
Source: PLoS One. 2022 Apr 21;17(4):e0266704. doi: 10.1371/journal.pone.0266704 (PMC9022879; doi:10.1371/journal.pone.0266704)
Supplement: S5 Fig — For the independent test data, these scatter plots compare the correlations in normal spleen SUVbw (column A, E, I), SUVlbm (column B, F, J), SUVbsa (column C, G, K) and SUVfdg (column D, H, L) measurements with weight (row A, B, C, D), height (row E, F, G, H) and age (row I, J, K, L). Note, spleen concentrations were not measured in the training cohort and played no part in determining the BHN function used to calculate these SUVfdg values. (PDF) [file pone.0266704.s005.pdf]

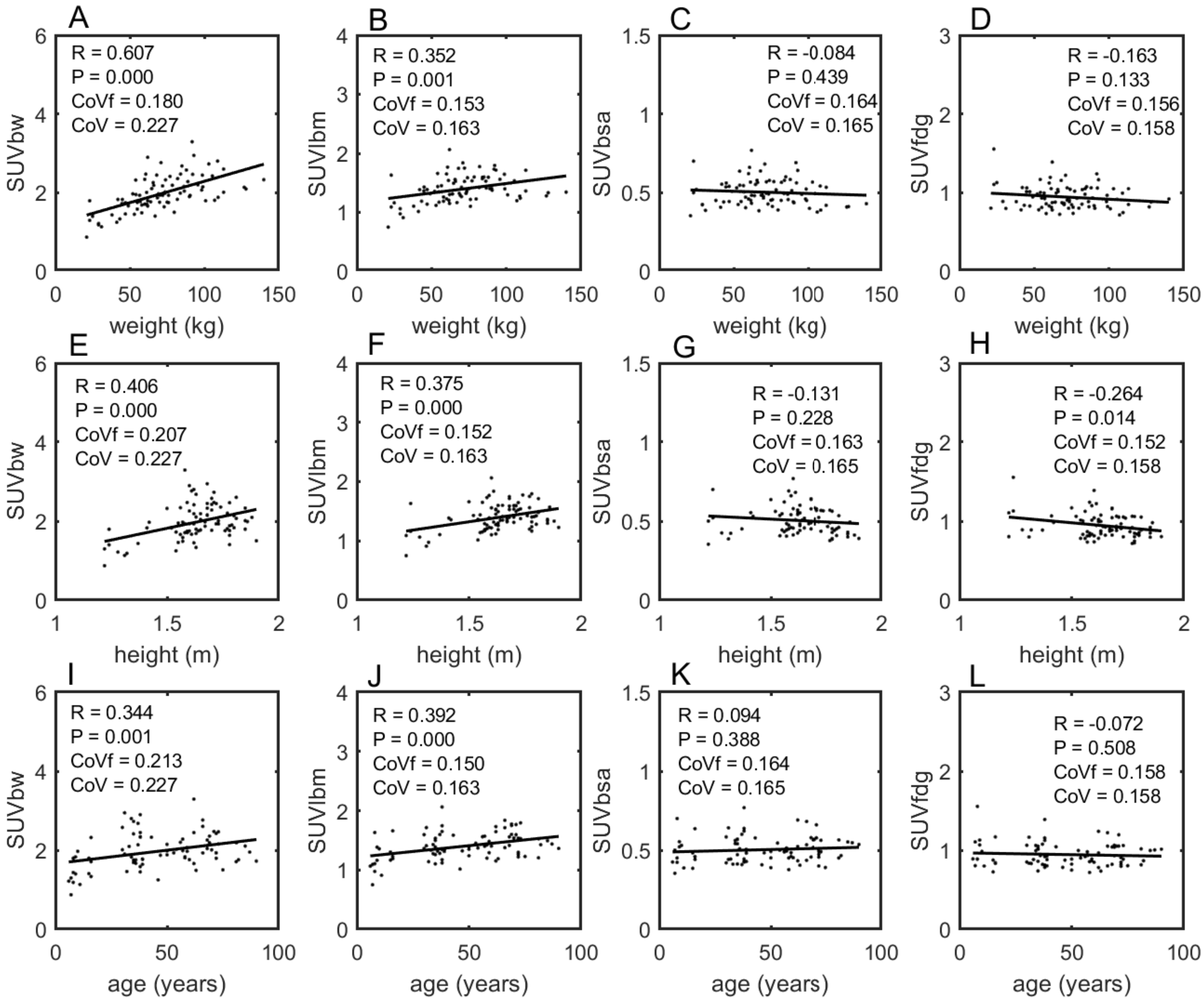

FIGURE S5. For the independent test data, these scatter plots compare the correlations in normal spleen SUVbw (column A, E, I), SUVlbm (column B, F, J), SUVbsa (column C, G, K) and SUVfdg (column D, H, L) measurements with weight (row A, B, C, D), height (row E, F, G, H) and age (row I, J, K, L). Note, spleen concentrations were not measured in the training cohort and played no part in determining the BHN function used to calculate these SUVfdg values.
