# Supplementary material for: SUVfdg: A standard-uptake-value (SUV) body habitus normalizer specific to fluorodeoxyglucose (FDG) in humans
Source: PLoS One. 2022 Apr 21;17(4):e0266704. doi: 10.1371/journal.pone.0266704 (PMC9022879; doi:10.1371/journal.pone.0266704)
Supplement: S6 Fig — For the brain-only independent test data, these scatter plots compare the correlations in normal frontal gray matter SUVbw (column A, E, I), SUVlbm (column B, F, J), SUVbsa (column C, G, K) and SUVfdg (column D, H, L) measurements with weight (row A, B, C, D), height (row E, F, G, H) and age (row I, J, K, L). Note, brain concentrations were not measured in the training cohort and played no part in determining the BHN function used to calculate these SUVfdg values. The lines and associated parameters seen in the legends were fitted to data from only the adult (>18 y) patients. The SUVfdg values suggest a significant difference in brain glucose metabolism between adult and pediatric populations. In all graphs, triangles depict male patients, x’s refer to female patients and o’s are children under the age of 18. (PDF) [file pone.0266704.s006.pdf]

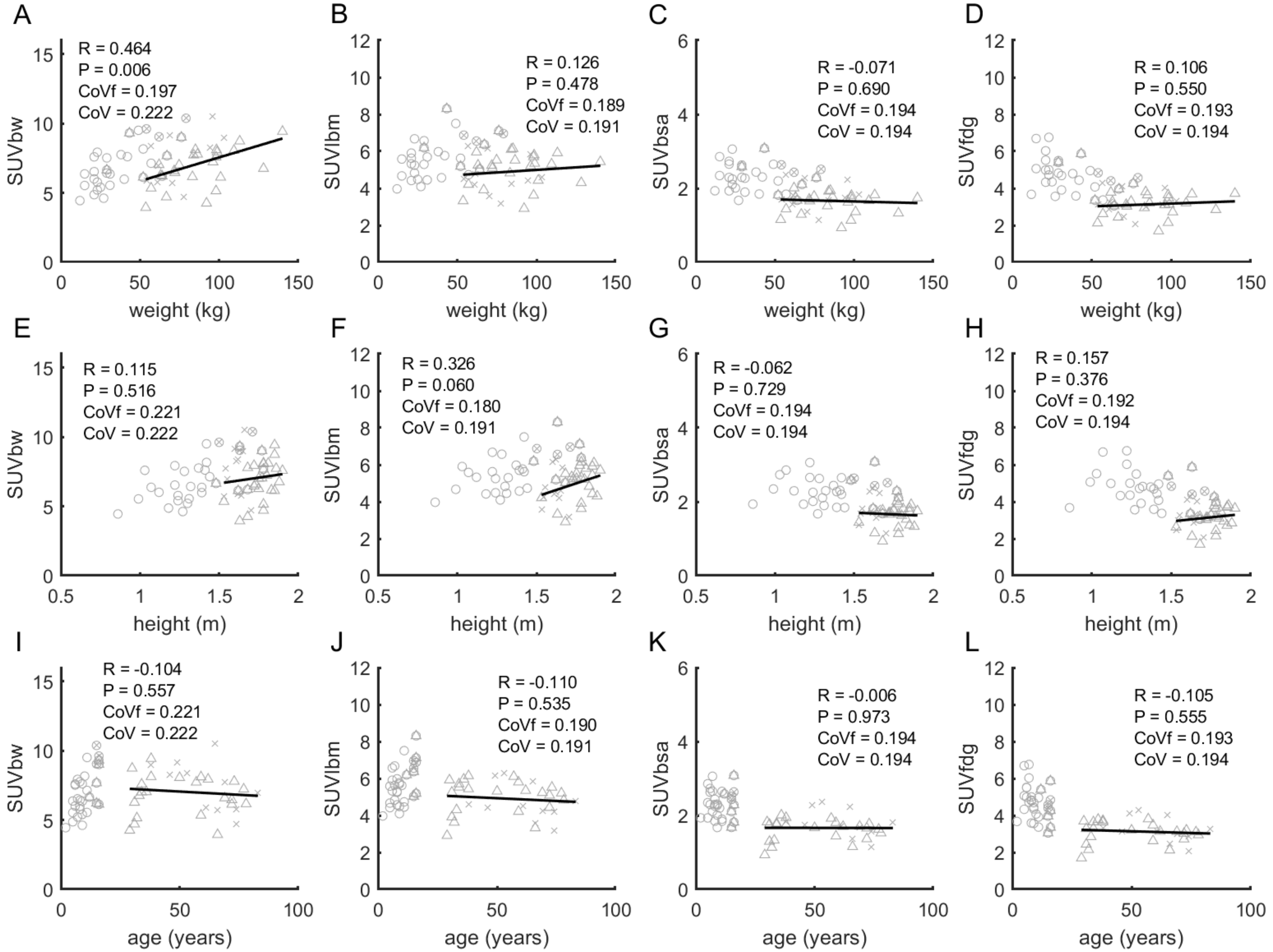

FIGURE S6. For the brain-only independent test data, these scatter plots compare the correlations in normal frontal gray matter SUVbw (column A, E, I), SUVlbm (column B, F, J), SUVbsa (column C, G, K) and SUVfdg (column D, H, L) measurements with weight (row A, B, C, D), height (row E, F, G, H) and age (row I, J, K, L). Note, brain concentrations were not measured in the training cohort and played no part in determining the BHN function used to calculate these SUVfdg values. The lines and associated parameters seen in the legends were fitted to data from only the adult (>18 y) patients. The SUVfdg values suggest a significant difference in brain glucose metabolism between adult and pediatric populations. In all graphs, triangles depict male patients, x's refer to female patients and o's are children under the age of 18.
